# Effects of Social Exclusion on Self-Evaluation: Domain Discrepancy Based on the Big Two Model

**DOI:** 10.3390/bs14090849

**Published:** 2024-09-20

**Authors:** Chao Zhang, Bing Chen, Yan Bao, Jiani He, Feng Wu, Yufang Zhao

**Affiliations:** 1 Faculty of Psychology, Southwest University, Chongqing 400715, China; chao0405@email.swu.edu.cn (C.Z.); baoyan@email.swu.edu.cn (Y.B.); chichi0327@email.swu.edu.cn (F.W.); 2 Center for Studies of Education and Psychology of Minorities in Southwest China, Southwest University, Chongqing 400715, China; jianihepsy@163.com

**Keywords:** social exclusion, self-evaluation, communion, agency, the Big Two

## Abstract

Prior studies have demonstrated the detrimental effects of social exclusion on an individual’s self-perception. Nonetheless, existing literature has predominantly focused on its impact on global self-esteem, often neglecting the nuanced effects of various reasons for social exclusion on distinct dimensions of self-evaluation, such as agency and communion. Based on the Big Two model perspective, the present research aims to examine the differential impacts of social exclusion on the distinct dimensions of self-evaluation, namely agency and communion, considering the reasons for social exclusion. It is hypothesized that social exclusion affects different facets of self-evaluation—agency and communion—depending on the context of exclusion. Study 1 identified negative correlations between perceived social exclusion and self-evaluation measures through questionnaires, including global self-esteem and the self-concepts of agency and communion, within a sample of 483 participants (283 females). Studies 2a (*n* = 93; 75 females) and 2b (*n* = 91; 66 females), which employed a recall paradigm to manipulate social exclusion in the domains of communion and agency, respectively, revealed that communion exclusion diminished communal self-evaluation, and agency exclusion significantly reduced both agentic and communal self-evaluation. These findings highlight the necessity of distinguishing various types of social exclusion and their distinct effects on the dimensions of self-concept. The research has important implications for the development of interventions aimed at enhancing individual psychological well-being and promoting inclusive social environments.

## 1. Introduction

“Know thyself,” the ancient Greek aphorism, is a seemingly simple but major concern across the life span that has inspired people to explore and understand themselves. In the process of acquiring and maintaining self-knowledge, people continuously assess and form opinions about their characteristics, abilities, behaviors, and overall self-perception, such as whether the self is intelligent, capable, or friendly, resulting in self-evaluation [1,2]. Possessing accurate and positive self-evaluation is highly significant for individuals, as it promotes self-development and self-improvement [3] and plays an important role in enhancing social adaptation and coordination of interpersonal relationships. As such, it helps in maintaining a healthy psychology and shaping correct behavioral patterns [4]. Nevertheless, humans are social in nature, and the self-concept is created and maintained in the course of social interaction. However, self-evaluation, an important component of self-concept [5], is subject to change as a result of social contextual factors such as social comparisons [6,7] and interpersonal relationships [8,9].

Social exclusion, as a negative experience that disrupts interpersonal connections when individuals are excluded from social interactions, groups, or communities, is likely to impact individuals’ self-evaluation. This multifaceted phenomenon can take various forms, including being ignored, rejected, or ostracized by others; can occur in various social situations [10]; and taps into fundamental human needs for belonging and social connection [11,12]. Being excluded by someone or a group may produce individuals’ concerns about whether the traits, personalities, or abilities they possess are attractive to their partner and may ultimately lead to a change in downward self-evaluation [13]. Many studies have confirmed that social exclusion is negatively correlated with self-esteem. When individuals are excluded, they may perceive it as a signal that they do not belong or are not valued by the social group, leading to thoughts of self-blame, self-doubt, negative self-attributions, a negative self-image, and diminished self-worth [14,15,16,17,18]. Therefore, the implications of social exclusion may include changes in self-evaluation and various behaviors that depend thereon.

In a social exclusion situation, people may change their own evaluation in terms of both agency and communion, because they typically evaluate themselves based on traits related to these fundamental dimensions [19,20,21]. Although both dimensions have slightly different names or labels across time and in different research contexts, such as warmth and competence [22,23], morality and competence [24], and socially and intellectually good/bad [25], they converge in their core content [26,27]. Furthermore, research on self-esteem has suggested that self-attitudes fall roughly into the two dimensions of communion and agency [28,29]. Communion and agency constitute the basic dimensions of self-evaluation. Most of the literature on social exclusion primarily focuses on changes in global self-evaluation or self-esteem following social exclusion; however, it has often overlooked distinctions in the specific content of the self-evaluation. Thus, this study aims to examine the effects of social exclusion on self-evaluation based on communal and agentic dimensions. Based on the Big Two model, in this study, we define agentic self-evaluation as an individual’s assessment of their qualities related to goal achievement (e.g., capable, assertive), while communal self-evaluation refers to an individual’s assessment of their qualities related to establishing and maintaining social relationships (e.g., friendly, honest).

Agency and communion reflect two core challenges in people’s lives: pursuing personal goals and belonging to a social group [30]. The first challenge induces people to build skills, develop competencies, strive to stand out, and get ahead, which are reflected in agentic characteristics and behavioral tendencies. The second challenge induces people to care for others, build connections, emphasize their commonalities, strive to fit in, and gain social acceptance, which is reflected in communal characteristics and behavioral tendencies [30,31]. The communion dimension emphasizes qualities relevant to social connection, including positive traits (e.g., friendly, kind) and negative traits (e.g., cool, dishonest). However, social exclusion undermines social connections, and social exclusion can be seen as a threat to communion [31]. In other words, communal traits can significantly impact social bonds and are necessary for individuals to connect with and be accepted by others. In turn, self-ratings of communal traits may be directly affected by social exclusion or rejection. The agency dimension refers to qualities relevant to goal achievement, including positive traits (e.g., intelligent, competent) and negative traits (e.g., passive, inefficient). Research revealed that negative personal events (e.g., achievement-related failure, the experience of failure at a task) diminish self-evaluation of agentic traits [32,33]. However, scant attention has been paid to whether social exclusion as a social event affects self-evaluation of agentic traits. According to the dual perspective model (DPM), self-perception (the actor perspective) is dominated by agency over communion [34]. Agentic traits are more easily accessible and receive more attention when perceiving the self. Furthermore, self-rated agency is strongly correlated with, and even a dominant predictor of, self-esteem and self-evaluation [35,36]. As mentioned, social exclusion leads to decreased self-esteem, and may further affect self-rated agency given the relationship between self-esteem and agency. Meanwhile, the need–threat model posits that social exclusion threatens two fundamental human needs: efficacy needs and relational needs [12]. The efficacy needs reflect the desire to establish and maintain a positive self-perception of one’s abilities, while the relational needs represent the desire to form and sustain harmonious, stable social connections [37]. These two dimensions of need delineate two critical aspects of self-worth, aligning with the connotations of agency and communion. When individuals experience exclusion, the threat to their efficacy and relational needs may negatively impact their self-evaluations in these respective domains. In conclusion, by integrating the agency and communion framework for self-evaluation into the need–threat model of social exclusion, this study attempts to disentangle the effects of social exclusion on agentic and communal self-evaluation, respectively.

In addition, different reasons for social exclusion may have distinct impacts on self-evaluation. Current paradigms for studying social exclusion in most of the empirical works, such as the cyberball and get acquainted paradigms, often involve pure and arbitrary exclusions, and subjects are never told why [38,39]. However, in real-life scenarios, social exclusion is not a capricious behavior that results from people’s impulsiveness [38]. People’s acceptance or rejection of others is typically based on their perceptions and judgments of the target [40]. Thus, although such manipulations without information on the reason for social exclusion would avoid the confounding influence of additional factors [38], they also ignore the complexity of social exclusion and may overlook the fact that the motivations and underlying reasons therefore could result in varying psychological effects. For example, some studies indicated that when social exclusion is based on other people’s preferences, participants’ self-esteem decreases significantly, although random exclusion has no impact thereon [41,42]. Rudert et al. (2023) [43] discovered that individuals engage in the social exclusion or rejection of others based on both communion (e.g., norm-violating) and agency (e.g., underperforming). Moreover, when individuals experience social exclusion, they think about the probable perceptions and judgments of excluders or rejecters of them in terms of communion and agency, and have different emotional reactions depending on their perceptions of why they are excluded or rejected [38]. As per the accessibility principle of cognitive and social psychology [44], the current representation of an object becomes relatively rich in negative content when individuals recall negative information about it, and equally rich in positive content when individuals recall positive information about it [36]. Following the accessibility logic, it can be inferred that when individuals perceive social exclusion that signaled either communion or agency as the reason for being excluded, the corresponding facets (communion or agency) of self-evaluation could become more responsive and susceptible to the effects of exclusion. Empirical research on this aspect is lacking, a shortcoming this study aims to address. Based on the previous review, it is hypothesized that social exclusion affects different facets of self-evaluation—agency and communion—depending on the context of exclusion. When individuals are excluded for reasons related to agency or communion, their self-evaluation in the corresponding domain may become more negative.

Understanding the effects of social exclusion on individuals’ self-evaluation is crucial for obtaining insights into the psychological consequences thereof and developing effective strategies to mitigate its negative effects. The Big Two model offers a powerful framework for understanding individual self-evaluation, distinguishing between the two fundamental dimensions of agency and communion. This allows for a deeper understanding of the multidimensional impact of social exclusion on self-evaluation. Additionally, agency and communion are also the two primary aspects through which individuals perceive the reasons for social exclusion [38,39], providing a valuable perspective for studying the effects of different types of social exclusion. Based on the Big Two model perspective, we conducted three studies to examine the effects of social exclusion on individuals’ self-evaluation of communion and agency and the potentially different evaluation patterns when social exclusion was due to the reasons of communion or agency. Specifically, Study 1 examined the relationship between social exclusion for no reason and self-ratings of communal traits and agentic traits using a questionnaire. The Rosenberg Self-Esteem Scale [45] was also included as a measure of global self-esteem, which represents an individual’s overall positive or negative attitude toward themselves. Studies 2a and 2b, respectively, manipulated communal reasons for exclusion and agentic reasons for social exclusion using the recall paradigm, that is, the participant is excluded for a lack of communion (Study 2a) or lack of agency (Study 2b). They then measured individuals’ self-evaluations on communal and agentic traits, as well as state self-esteem (global self-evaluation).

## 2. Study 1

### 2.1. Materials and Methods

#### 2.1.1. Participants

This study was conducted using an online survey platform (www.wjx.com). In total, 483 college students from 2 universities in southwest China completed the online questionnaire survey. The sample included 200 male participants and 283 female participants, with an average age of 21.17 years (*SD* = 1.90, range 18 to 28). Participants were compensated for their participation. This study was approved by the ethics committee of the university (IRB No. Approved: H23120).

#### 2.1.2. Measures

Social Exclusion: The Social Exclusion Questionnaire for College Students [46] was used to measure experiences of social exclusion. This questionnaire comprises 19 items categorized into 2 dimensions: direct exclusion and indirect exclusion. Sample items include the following: “I am kept away from others when playing together” and “Others do not greet me even though we know each other.” The items were rated on a 5-point Likert scale from 1 (hardly ever) to 5 (almost always). A high score indicates more experiences of being excluded by others. Cronbach’s α was 0.96 in this study.

Global Self-esteem: We measured global self-esteem using the Chinese version of the Self-Esteem Scale [47], which comprises 10 items (e.g., “On the whole, I am satisfied with myself”) and utilizes a 4-point Likert scale ranging from 1 (strongly disagree) to 4 (strongly agree). Higher scores indicate a higher level of self-esteem. The Cronbach’s α was 0.89 in this study.

Agency and Communion: The self-evaluations of agency and communion were measured via 20 adjectives describing traits, with an equal number of positive and negative items. Of the items, 10 assessed agency (e.g., competent, clever, unintelligent, insecure), and the other 10 measured communion (e.g., kind, warm, unfriendly, offish). These traits were selected from an established Chinese Adjective Words System for Fundamental Dimensions of Social Cognition [48], which were carefully balanced for familiarity, word frequency, and their agency versus communion-relatedness. Participants scored each item on a 7-point Likert scale ranging from 1 (definitely does not apply to me) to 7 (definitely applies to me). After reversing the scores of the negatively worded items, we averaged the items for each dimension and used them to index self-evaluations of agency (Cronbach’s α = 0.86) and communion (Cronbach’s α = 0.86).

### 2.2. Results

#### 2.2.1. Common Method Bias Test

Harman’s single-factor test was conducted to assess common method bias. The results revealed that seven factors were extracted with eigenvalues greater than 1, and the first factor could explain 35.71% of the variance. Consequently, common method bias is not a major concern in the study.

#### 2.2.2. Correlation and Regression Analysis

The means, standard deviations, and correlations of the main variables are presented in Table 1. Social exclusion was negatively correlated with global self-esteem (*r* = −0.53, *p* < 0.001), agency (*r* = −0.41, *p* < 0.001), and communion (*r* = −0.48, *p* < 0.001). In addition, global self-esteem, agency, and communion were all positively correlated with each other.

Next, we conducted separate regression analyses for global self-esteem, agency, and communion, with social exclusion as the predictor, controlling for age and gender. The results showed that social exclusion significantly negatively predicted global self-esteem (*β* = −0.55, *t* = −14.51, *p* < 0.001), agency (*β* = −0.44, *t* = −11.29, *p* < 0.001), and communion (*β* = −0.50, *t* = −12.99, *p* < 0.001). Furthermore, the regression coefficients for predicting global self-esteem, communion, and agency from social exclusion were tested separately for males and females. Among males, the regression coefficients for global self-esteem (*β* = −0.60, *t* = −10.53, *p* < 0.001), agency (*β* = −0.53, *t* = −8.72, *p* < 0.001), and communion (*β* = −0.52, *t* = −8.59, *p* < 0.001) were all significantly predicted by social exclusion. Likewise, in females, the regression coefficients for self-esteem (*β* = −0.51, *t* = −10.05, *p* < 0.001), agency (*β* = −0.40, *t* = −7.34, *p* < 0.001), and communion (*β* = −0.50, *t* = −8.79, *p* < 0.001) were also significantly predicted by social exclusion.

Consistent with previous literature, the findings of Study 1 indicated that social exclusion was negatively related to global self-esteem. Furthermore, as expected, social exclusion was negatively associated with both agency and communion. This means that chronic exclusion experiences in real life can decrease individuals’ evaluations of their own agency and communion. Considering that individuals are not excluded or rejected without reason in real life, we manipulated communion and agency exclusion separately based on the perspective of the Big Two model in Studies 2a and 2b to examine their effects on self-evaluation.

## 3. Study 2a

In Study 2a, we manipulated communion exclusion to explore the influence of social exclusion on communal and agency-based self-evaluation.

### 3.1. Materials and Methods

#### 3.1.1. Participants

This study involved 93 participants from a university in Chongqing, China (75 females and 18 males, *M*_age_ = 20.71 years, *SD*_age_ = 2.11 years), after excluding 2 participants who did not correctly recall the exclusion experience as required (excluded due to communion aspects). A sensitivity power analysis for a three-way-mixed analysis of variance with G*Power 3.1.9.7 showed that a sample of 93 participants yielded a small to medium effect size (*f* = 0.122) with a power of 0.80 (α = 0.05). Participants provided their informed consent and received monetary compensation for their participation. The study was approved by the ethics committee of the university (IRB No. Approved: H23120).

#### 3.1.2. Procedure

Upon arriving at the laboratory, participants learned the research involved several unrelated components. First, they underwent a communion exclusion manipulation in which they recalled and wrote about a past experience of exclusion (or a neutral control topic) [49]. By random assignment, participants in the exclusion condition were asked to recall and write a past experience of being excluded or rejected due to communion. To assist participants in accurately recalling their experience, we provided them with an instance where the protagonist was excluded by others due to being questioned about their communion. By comparison, those in the neutral control condition were asked to recall and write about the activities they engaged in on a typical day from the past week. Afterward, as an indicator of their feelings of exclusion, participants rated three statements (e.g., “I was excluded in the experience”) on a 7-point Likert scale (1 = strongly disagree, 7 = strongly agree). The scores of the three statements (Cronbach’s α = 0.94) were averaged to check the effectiveness of the manipulation.

Next, the participants completed the Self-Esteem Scale and a self-evaluation questionnaire based on agency and communion. The self-evaluation questionnaire, which is based on agency and communion, included 20 traits for communion (e.g., “kind” and “unfriendly”) and 20 traits for agency (e.g., “clever,” and “unintelligent”) selected from the well-established Chinese Adjective Words System for Fundamental Dimensions of Social Cognition [48]. Moreover, these trait words were balanced for positive/negative valence, familiarity, and word frequency, similar to Study 1. For each item, participants were asked to evaluate themselves compared to their peers of the same gender on a 9-point Likert scale (1 = bottom 10%, 5 = middle 50%, 9 = top 10%). In addition, we used the same Self-Esteem Scale (Cronbach’s α = 0.88) as in Study 1 to measure participants’ self-esteem state. By adapting the instructions, they were asked to respond to each item based on their current feelings. A final thank you and debriefing were given to the participants.

### 3.2. Results

Manipulation Check: An independent sample *t*-test was performed to determine the effectiveness of the exclusion manipulation. The results indicated that participants in the exclusion condition (*M* = 5.67, *SD* = 0.81) reported being more excluded than those in the control condition (*M* = 2.19, *SD* = 0.99, *t* = 18.38, *p* < 0.001, Cohen’s *d* = 3.847), which indicated that manipulation was effective.

State Self-esteem: A one-way analysis of variance, which was applied to self-esteem state, showed no significant differences in the scores of self-esteem state between the exclusion and control conditions (*F* (1, 91) = 0.05, *p* = 0.82, η^2^*_p_* = 0.001). This indicates that the manipulation of exclusion did not affect participants’ self-esteem state.

Agency: We conducted a mixed-factor analysis of variance on agency, with exclusion (exclusion condition vs. control condition) as a between-subjects variable and trait valence (positive agency vs. negative agency) as a within-subjects variable. The results revealed that the main effect of trait valence was significant (*F* (1, 91) = 94.70, *p* < 0.001, η^2^*_p_* = 0.510), indicating that scores for positive agency (*M* = 5.63, *SD* = 1.02) were significantly higher than those for negative agency (*M* = 3.80, *SD* = 1.10). However, the main effect of exclusion was not significant (*F* (1, 91) = 0.23, *p* = 0.63, η^2^*_p_* = 0.003), and neither was the interaction between exclusion and trait valence (*F* (1, 91) = 0.04, *p* = 0.84, η^2^*_p_* < 0.001).

Communion: Similarly, a 2 (exclusion) × 2 (trait valence) mixed analysis of variance was conducted, with communion as the dependent variable. This analysis revealed a significant main effect of trait valence (*F* (1, 91) = 406.09, *p* < 0.001, η^2^*_p_* = 0.82), but no main effect of exclusion (*F* (1, 91) = 2.62, *p* = 0.11, η^2^*_p_* = 0.028). Importantly, a significant interaction between exclusion and trait valence was observed (*F* (1, 91) = 7.71, *p* = 0.007, η^2^*_p_* = 0.078). Simple-effects analyses revealed that participants in the exclusion condition reported higher ratings of negative communion (*M* = 3.47, *SD* = 1.11) than those in the control condition (*M* = 2.85, *SD* = 0.87, *F* (1, 91) = 9.12, *p* = 0.003, η^2^*_p_* = 0.091). However, there was no significant difference in ratings of positive communion between participants in either the exclusion or control conditions (*F* (1, 91) = 1.08, *p* = 0.30, η^2^*_p_* = 0.012). When controlling for gender through an analysis of covariance, the interaction effect between exclusion and trait valence remained significant (*F* (1, 91) = 7.42, *p* = 0.008, η^2^*_p_* = 0.076). Participants in the exclusion condition reported higher ratings of negative communion than those in the control condition.

## 4. Study 2b

In Study 2b, we manipulated agency exclusion to examine the influence of social exclusion on communal and agency-based self-evaluation.

### 4.1. Materials and Methods

#### 4.1.1. Participants

This study involved 91 participants from a university in Chongqing, China (66 females and 25 males, *M*_age_ =20.76 years, *SD*_age_ = 1.74 years), after excluding 4 participants who did not correctly recall the exclusion experience as required (excluded due to agency aspects). A sensitivity power analysis for a three-way-mixed analysis of variance with G*Power 3.1.9.7 showed that a sample of 91 participants yielded a small to medium effect size (*f* = 0.123), with a power of 0.80 (α = 0.05). Participants provided their informed consent and received monetary compensation for their participation. The study was approved by the ethics committee of the university (IRB No. Approved: H23120).

#### 4.1.2. Procedure

As in Study 2a, upon their arrival at the laboratory, participants learned that the research involved several unrelated components. Participants were randomly assigned to either the agency exclusion or neutral control condition. Participants in the agency exclusion condition were asked to recall and write about a past experience of being excluded or rejected owing to agency. To assist participants in recalling their experience accurately, we provided them with an instance where the protagonist was excluded by others due to being questioned about their agency. Participants in the neutral control condition completed the same tasks as those in Study 1. After the recalling tasks, all participants responded to three statements (e.g., “I was excluded in the experience”) on a 7-point Likert scale. The scores of the three statements (Cronbach’s α = 0.93) were averaged to check the effectiveness of the manipulation.

Next, participants completed the Self-Esteem Scale (Cronbach’s α = 0.88) and a self-evaluation questionnaire based on agency and communion, which were the same as in Study 2a. A final thank you and debriefing were given to the participants.

### 4.2. Results

Manipulation Check: Participants in the exclusion condition (*M* = 5.58, *SD* = 0.70) felt more excluded than those in the control condition (*M* = 2.23, *SD* = 0.99, *t* = 18.67, *p* < 0.001, Cohen’s *d* = 2.829). Therefore, the exclusion manipulation was effective.

State Self-esteem: The results of the one-way analysis of variance revealed no significant differences in the scores of the self-esteem state between participants in the exclusion and control conditions (*F* (1, 89) = 3.37, *p* = 0.07, η^2^*_p_* = 0.036).

Agency: We conducted a 2 (exclusion: exclusion condition vs. control condition) × 2 (trait valence: positive agency vs. negative agency) mixed analysis of variance on agency. The results revealed that the main effect of trait valence was significant (*F* (1, 89) = 108.07, *p* < 0.001, η^2^*_p_* = 0.548), indicating that scores for positive agency (*M* = 5.55, *SD* = 0.84) were higher than those for negative agency (*M* = 3.89, *SD* = 1.03). The main effect of exclusion was not significant (*F* (1, 89) = 0.07, *p* = 0.80, η^2^*_p_* = 0.001). Moreover, the results showed that the interaction effect was significant (*F* (1, 89) = 9.10, *p* = 0.003, η^2^*_p_* = 0.093). Participants in the exclusion condition reported higher ratings of negative agency (*M* = 4.12, *SD* = 1.00) than those in the control condition (*M* = 3.67, *SD* = 1.03, *F* (1, 89) = 4.48, *p* = 0.04, η^2^*_p_* = 0.048). However, participants in the exclusion condition reported lower ratings of positive agency (*M* = 5.29, *SD* = 0.82) than those in the control condition (*M* = 5.80, *SD* = 0.79, *F* (1, 89) = 9.06, *p* = 0.003, η^2^*_p_* = 0.092). When controlling for gender through an analysis of covariance, the interaction effect between exclusion and trait valence remained significant (*F* (1, 89) = 9.09, *p* = 0.003, η^2^*_p_* = 0.094).

Communion: A 2 (exclusion: exclusion condition vs. control condition) × 2 (trait valence: positive agency vs. negative agency) mixed analysis of variance, with communion as the dependent variable, yielded a main effect of trait valence (*F* (1, 89) = 409.72, *p* < 0.001, η^2^*_p_* = 0.822), such that positive agency (*M* = 6.15, *SD* = 0.92) was always rated higher than negative agency (*M* = 3.11, *SD* = 0.95). The main effect of exclusion was not significant (*F* (1, 89) = 0.12, *p* = 0.73, η^2^*_p_* = 0.001). Moreover, there was an interaction between exclusion and trait valence (*F* (1, 89) = 5.28, *p* = 0.02, η^2^*_p_* = 0.056). The simple-effects test revealed that participants in the exclusion condition rated positive communion lower (*M* = 5.96, *SD* = 0.94) than did those in the control condition (*M* = 6.35, *SD* = 0.87, *F* (1, 89) = 4.16, *p* = 0.04, η^2^*_p_* = 0.045). However, ratings of negative communion did not differ between the participants in either the exclusion or control condition (*F* (1, 89) =2.33, *p* = 0.13, η^2^*_p_* = 0.026). When controlling for gender through an analysis of covariance, the interaction effect between exclusion and trait valence remained significant (*F* (1, 89) = 5.06, *p* = 0.03, η^2^*_p_* = 0.054). The results were largely consistent.

## 5. Discussion

From a Big Two model perspective, this research investigated the impact of social exclusion on individual self-evaluation through three studies. The results indicated that general perceptions of exclusion are significantly negatively correlated with global self-esteem and communal and agentic self-evaluation. Moreover, communion exclusion significantly reduced communal self-evaluation, while agency exclusion reduced both agentic and communal self-evaluation. However, neither form of exclusion significantly impacted the self-esteem state. These findings reveal the multidimensional impact of social exclusion on individual self-evaluation and highlight the specific patterns of social exclusion in different fields of self.

Social exclusion impacts individuals’ self-evaluation, and according to the need–threat model, is widely considered a threat to individuals’ fundamental psychological needs [50]. The fulfillment of these needs is crucial for individual psychological health and positive self-evaluation [51]. When these needs are denied, individuals may feel profound psychological distress and a decrease in self-worth. This study revealed that social exclusion undermines individuals’ global self-esteem and self-evaluation by threatening their sense of belonging and self-worth. Furthermore, general social exclusion negatively influences self-evaluation in the dimensions of both communion and agency within the framework of the Big Two model.

Different patterns of self-evaluation emerge in both communion and agency based on the reasons for social exclusion. Specifically, social exclusion in the communal domain primarily negatively affects communal self-evaluation, which means an impact in the same domain. In contrast, social exclusion in the agentic domain negatively impacts both agentic and communal self-evaluation, thereby extending its influence to another domain. As we know, the self is a complex polyhedron composed of various substructures, and the aspects of the self-concept may be activated or elicited by specific experiences, events, or situations [52]. Based on the tenet of accessibility of cognitive and social psychology [36,44], when individuals are excluded due to reasons related to communion or agency, the negative communion or agency content of one’s self-perception may become more salient or accessible. Therefore, individuals begin to question their self-evaluation of communion or agency.

Noteworthy is that agency exclusion has a generalized effect on self-evaluation that extends to the communal dimension, which indicates the existence of a “dominating effect of agency” in self-evaluation. According to the DPM [34], when individuals engage in self-perception (actor perspective), they tend to place more importance on the agentic dimension. A person’s global self-esteem is more related to agency than communion, and agency seems to be more important for global self-evaluation [53]. Given the importance of agency in self-perception, social exclusion based on agentic reasons may negatively impact an individual’s overall self-evaluation or self-worth. For example, research has shown that a threat to a person’s agency leads to more identity threats, which reflect how negatively a person thinks about himself [53]. This negative self-view may include perceptions of autonomy and self-efficacy and assessments of social status and social value within relationships. This may lead to the negative generalized impact of agency exclusion on the evaluation of the communal dimension. Simultaneously, due to the importance of agency to the overall self, when experiencing communion exclusion, the reasons for being excluded are clearly focused on communion. Motivated by self-protection and the need to maintain core self-esteem, individuals are unlikely to interpret social exclusion as a doubt in their competence or autonomy. Therefore, their agency evaluation is likely to remain unaffected.

### 5.1. Theoretical Contributions and Practical Implications

We believe that the findings of this study have important theoretical contributions. First, the research expands the literature on social exclusion, revealing differences in evaluation patterns in this field. By distinguishing communal and agentic self-evaluation, the study provides a more nuanced understanding of how social exclusion impacts different dimensions of self-concept. Second, this study highlights the relationship between agency and communion. In self-evaluation, especially negative ones, the dominating effect of agency may occur, where other’s denial of one’s agency can bring disaster to the self-evaluations of agency and communion. The findings of this study suggest that their relationship may be moderated by social feedback (e.g., social exclusion). In addition, by integrating the Big Two model with theories on social exclusion, the present study extends the need–threat model. The need–threat model illustrates that social exclusion threatens basic psychological needs such as self-esteem, belongingness, and a sense of control [50]. However, this model fails to consider how different underlying reasons for social exclusion, such as agency or communion, might threaten these needs and thus produce subsequent changes in self-evaluation.

Moreover, our research has practical implications for the design of social policies and educational programs to mitigate the detrimental effects of social exclusion. The findings suggest that interventions should consider both communal and agentic aspects of an individual’s needs when addressing the aftermath of social exclusion, offering a more holistic approach to psychological support. Schools and universities can develop programs that specifically address different forms of exclusion among students. Programs that encourage group cooperation and social inclusion could counteract the effects of communal exclusion, while initiatives that provide leadership opportunities and decision-making roles can help students excluded due to agentic reasons rebuild their self-efficacy.

### 5.2. Limitations and Future Research Directions

While this research provides valuable insights, it has a few shortcomings, which can be improved on in future research. This study employed a recall paradigm to manipulate social exclusion in different domains, but the completeness of recalled exclusion events may be affected by variations in participants’ memory abilities. Future studies could employ more ecologically valid methods, such as virtual reality, to enhance external validity. Considering the inconsistent findings regarding the impact of social exclusion on self-esteem [54], future research can employ diverse measurement tools and longitudinal designs to examine how agency and communion-based exclusion affect immediate changes in self-esteem state. As agency can be further distinguished into the facets of agency–competence (AC) and agency–assertiveness (AA), and communion into communion–warmth (CW) and communion–morality (CM) [35,53], future studies could explore the role of these sub-dimensions in the context of social exclusion. Moreover, future research should explore the effects of different types of social exclusion on multidimensional self-evaluation in various cultural contexts, because cultural differences in norms, values, and interpersonal relationships can shape how social exclusion is experienced and its subsequent impact on self-evaluation [55,56]. Understanding these cultural nuances can provide a more comprehensive perspective on social exclusion and inform culturally sensitive interventions.

## 6. Conclusions

This research establishes a robust link between social exclusion and decreased self-evaluation, delineating the effects on global self-esteem and the self-concept aspects of agency and communion. Social exclusion broadly undermines global self-esteem, and communal and agentic self-evaluation. Communion exclusion affects communal self-evaluation, while agency exclusion has a more generalized impact, reflecting its broader implications for both agentic and communal self-evaluation. By distinguishing domain-specific exclusion, this study advances our understanding of how different types of exclusion uniquely influence self-evaluation, which is crucial for developing targeted interventions to mitigate the adverse effects of social exclusion.

## Figures and Tables

**Table 1:** Descriptive statistics and correlations among variables (*n* = 483).

Variables	*M*	*SD*	1	2	3	4
1. Social exclusion	1.65	0.72	1			
2. Global self-esteem	3.27	0.56	−0.53 **	1		
3. Agency	5.24	1.03	−0.41 **	0.72 **	1	
4. Communion	5.76	0.86	−0.48 **	0.60 **	0.67 **	1

Note: ** *p* < 0.001 (two tails).

## Data Availability

The data that support the findings of this study are available from the corresponding author upon reasonable request.
